# The impact of environmental pollution on China's economic growth from the perspective of health damage

**DOI:** 10.3389/fpubh.2025.1562342

**Published:** 2025-04-24

**Authors:** Hao Li, Zihan Yang, Jiahui Chen

**Affiliations:** ^1^School of Business, Xinyang Normal University, Xinyang, China; ^2^Research Institute of the Economic and Social Development in the Dabie Mountains, Xinyang, China

**Keywords:** environmental pollution, health damage, simultaneous equations model, economic growth, endogenous growth model

## Abstract

**Introduction:**

Achieving an optimal economic growth rate is a cornerstone of high-quality development. Among the diverse factors influencing economic growth, environmental pollution and health-related issues have emerged as critical concerns in recent years.

**Methods:**

This study develops a five-sector endogenous growth model that incorporates the effects of environmental pollution on health. The model theoretically examines the mechanisms through which environmental pollution and health jointly influence economic growth. Using panel data from 285 Chinese cities spanning 2002 to 2022, we empirically analyze the impact of environmental quality and health levels on economic growth, based on comprehensive measurements of these variables in Chinese urban areas.

**Results:**

The findings indicate that environmental quality and health are key drivers of economic growth. Specifically, environmental pollution not only directly hampers economic growth but also indirectly impedes it by degrading public health.

**Discussion:**

These results underscore the importance of implementing robust environmental pollution control policies and improving public health standards. By reducing the adverse effects of environmental pollution on health, policymakers can mitigate the risk of environmental and health deterioration, thereby safeguarding China's pursuit of high-quality economic development.

## 1 Introduction

China's economic transformation over the past four decades, driven by reform and opening-up policies, has positioned it as the world's second-largest economy and a primary engine of global growth, contributing nearly 30% to worldwide economic expansion. However, this “growth miracle” has relied on an unsustainable model marked by high resource consumption, pollution-intensive production, and low efficiency (1). The resulting environmental degradation has imposed severe health costs, undermining human capital—a critical determinant of economic productivity (2). Mounting evidence reveals that environmental pollution exacerbates health disparities, reduces labor productivity, and constrains long-term economic sustainability (3). For instance, air and water pollution correlate with rising incidences of respiratory diseases, chronic conditions, and mental health burdens, disproportionately affecting vulnerable populations (4). Such health deterioration not only escalates public healthcare expenditures but also diminishes workforce efficiency, creating a feedback loop that jeopardizes both environmental and economic resilience (5). Addressing these intertwined challenges aligns with China's national strategy of “Beautiful China,” which prioritizes ecological conservation and public health as pillars of high-quality development. Yet, the mechanisms linking pollution, health, and economic outcomes remain underexplored. This study aims to unravel these complexities, providing empirical insights to reconcile environmental sustainability with economic growth—a prerequisite for achieving the dual goals of health equity and ecological civilization.

## 2 Literature review and research hypotheses

In the existing research on the relationship between environmental pollution and economic growth, most studies have used the Environmental Kuznets Curve as an analytical framework. The studies have found that the relationship between environmental pollution and economic growth exhibits a bidirectional “U-shaped” or “inverted U-shaped” pattern (6). This relationship is primarily influenced by regional development levels, the intensity of environmental policies, and the level of technological progress (1). On one hand, environmental pollution directly inhibits sustainable economic development through resource depletion and ecological degradation (7). On the other hand, high-pollution industries, particularly during the initial stages of industrialization, promote economic growth through scale expansion, externalization of environmental impacts, job creation, and tax contributions (8). This often creates a path dependency of “first polluting, then regulating,” despite potentially triggering environmental degradation and economic unsustainability in the long run (9).

Research on the relationship between health and the economy primarily relies on Grossman's health capital theory, utilizing the Grossman health demand model for analysis. This theory posits that health levels indirectly affect economic development by influencing labor productivity, working years, and healthcare expenditures (10). Additionally, scholars have focused on the economic costs associated with the disease burden of health issues. Economic assessments of health damages often employ the human capital approach (HCA) and the willingness-to-pay method (WTP) (11). Furthermore, improvements in public health can unleash potential for economic growth. Some studies using Computational General Equilibrium (CGE) models have found that a 1% increase in health investment can boost long-term GDP growth by 0.3% to 0.5% (12).

In the study of the relationship between environmental pollution, health, and economic growth, health often serves as an intermediary variable for mechanism analysis. For instance, environmental pollution forms a transmission chain of “pollution → disease → loss of labor → economic recession” through health damage (5). Air pollution, for example, leads to an increase in the incidence of respiratory diseases, resulting in an increase in the absentee rate of manufacturing and service industries by 3–5%, especially for outdoor workers (3).

In the existing studies, health is often conceptualized as healthy human capital in theoretical analyses, emphasizing the direct contribution of healthy human capital to economic growth while neglecting the indirect effects of environmental pollution on economic growth via its impact on health (13). In the empirical analysis, changes in the ramifications of health damage caused by environmental pollution on economic growth often go unnoticed. While a few studies have considered these ramifications, most of them adopt a straightforward approach by using the level of health or a proxy variable for health damage caused by environmental pollution, inferred through subjective judgments and assigned a specific proportion (14). These studies implicitly assume the existence of a causal relationship between environmental pollution and health damage, but they tend to overlook or minimize the indirect influence of such health damage on economic growth. Consequently, they may not truly reflect the actual impact of environmental pollution-induced health damage on economic growth (14).

To address the shortcomings identified in prior literature, this study aims to enhance understanding in two primary domains: firstly, based on the four sector endogenous economic growth model under environmental constraints, we added the health sector and constructed a five-sector endogenous economic growth model of the impact of environmental pollution on economic growth from the perspective of health damage. We have included the factors that influence economic growth due to health damage caused by environmental pollution in our research scope, in order to analyze the changes in the contribution of health affected by environmental pollution to economic growth through comparative analysis. This five-sector endogenous economic growth model demonstrates that environmental pollution not only directly impacts economic growth but also indirectly affects it through its influence on health. This influence is mediated through various channels, including the accumulation of human capital, the advancement of technological research, development and innovation, adjustments in labor supply, variations in savings and consumption patterns, and fluctuations in public health spending. Second, focusing on 285 municipal administrative units from 2002 to 2022 as the empirical subjects and leveraging a multifaceted empirical analysis approach grounded in theoretical reasoning, this research delves into both the direct and indirect effects of environmental pollution and resultant health issues on economic growth.

Based on the above analysis, we propose the following hypotheses:

Research Hypothesis 1: environmental pollution and health are crucial determinants of economic growth.
Research Hypothesis 2: the impact of environmental pollution on economic growth is context-dependent and may vary under different circumstances.
Research Hypothesis 3: the interplay between environmental pollution, health, and economic growth leads to shifts in their respective influences on the overall economic development.

## 3 Theoretical studies

### 3.1 Mechanistic analysis of the impact of environmental pollution on economic growth from the perspective of health damage

#### 3.1.1 Health, education, human capital accumulation, and economic growth

Environmental pollution-induced health damage is detrimental to educational production and the accumulation of human capital. However, environmental policies can influence education, thus indirectly contributing to economic growth (15). An increase in life expectancy enhances the duration of the utilization of knowledge and skills acquired by individuals, leading to a corresponding rise in productive capacity and efficiency, thereby indirectly promoting economic growth (16). Consequently, enhancing the health of individual residents not only affects their learning abilities, cultural qualities, technical skills, and production processes, but also improves the efficiency of physical capital utilization and the productivity of society as a whole, ultimately driving economic growth (17). Health and education are the two most critical components of human capital. The impact of environmental pollution on human capital accumulation is primarily felt through its effect on health. Subsequently, this affects the education of individuals, thereby indirectly influencing the overall level of human capital accumulation and economic growth.

#### 3.1.2 Health, labor supply, labor productivity and economic growth

Based on the human capital theory, workers in good health are not only able to maintain their physical and mental wellbeing, thus enhancing their work efficiency, but also capable of handling higher work intensities and fast-paced work styles. This, in turn, improves the labor supply for the entire society, boosts overall productivity, and ultimately promotes economic growth (15). Conversely, the damage to health caused by environmental pollution has a significant impact on labor supply and labor productivity (5).

To explore the effects of environmental pollution-induced health damage on labor supply, this paper introduces several assumptions. Firstly, the population is exogenous, with a total regional population denoted as *N* and a regional labor force population as *L*. The non-labor force population, which excludes participation in local work due to factors such as workers' age, health damage caused by environmental pollution leading to a decrease in labor supply, or worker migration due to environmental pollution, is represented as (*N* − *L*). The share of the regional non-labor force population in the total regional population is *η*(0 < *η* < 1) denoted as *η* = (*N* − *L*)/*N*. Secondly, this article relaxes the assumption that the entire population consists of the labor force and that the labor force growth rate is zero. Instead, it assumes that the population growth rate is $n=\overset{•}{N}/N$, the labor force growth rate is $g_{L}=l=\overset{•}{L}/L$, and the growth rate of the non-labor force proportion is $\sigma =\overset{•}{\eta }/\eta$. Furthermore, this article posits that the relationships between the growth rates of the labor force, population, non-labor force proportion in the region, and their respective growth rates are as follows:


(1) $$\begin{array}{l} \overset{•}{\eta }\text{=}\frac{nN-lL}{N+nN}-\eta =\sigma \eta ,\Rightarrow l=\frac{n-\eta -n\eta -\eta \sigma \left(1+n\right)}{1-\eta } \end{array}$$

As shown in Equation 1, changes in the population growth rate, the share of the non-labor force, and the growth rate of the non-labor force impact the labor force growth rate:


(2) $$\begin{array}{l} \frac{\partial l}{\partial n}=\frac{1-\eta -\eta \sigma }{1-\eta },\frac{\partial l}{\partial \eta }=-\frac{1+\sigma \left(1+n\right)}{{\left(1-\eta \right)}^{2}},\frac{\partial l}{\partial \sigma }=\frac{\eta \left(1+n\right)}{\eta -1} \end{array}$$

The impact of health deterioration caused by environmental pollution, including the effects of the population's age structure, can result in an increase in the proportion of the non-labor force. This increase subsequently exerts a negative influence on the labor force growth rate.

To simplify the model, let's denote the regional non-labor force population by *W* = (*N* − *L*), and its growth rate by $\sigma =\overset{•}{W}/W-\overset{•}{L}/L$. The model then describes the relationship as follows:


(3) $$\sigma =\frac{\overset{•}{W}}{W}-\frac{\overset{•}{L}}{L}=\frac{n-l}{\eta }\Rightarrow g_{L}=l=n-\sigma \eta$$

Comparison of Equations 1–3 reveals that in Equation 3, the growth rate of the total population has a positive impact on the growth rate of the labor force population. Furthermore, the product of the growth rate of the non-labor force share and the non-labor force share itself exerts a negative influence on the growth rate of the labor force population.

#### 3.1.3 Health, technological innovation, and economic growth

Technological innovation is dependent on human beings for its implementation. The level of health and education in human capital directly affects the efficiency of technological innovation. Enhanced health facilitates the upgrading of the human capital structure, and an increase in the proportion of senior human capital can effectively drive the development, absorption, digestion, and application of new technologies. This, in turn, promotes economic growth (18). Environmental pollution causes health damages that not only result in increased internal governance costs and external prevention costs but also lead to insufficient funds for technological innovation. This subsequently affects the labor force's ability to receive education and skills training, as well as the time allocated to technological innovation by the labor force, the improvement of production processes, and the application of production technology (19). The reduction in the normal inflow of highly skilled labor within a region and undoubtedly inhibits the region's firms' green innovation capacity (20). On the other hand, technological innovation can compensate for the reduction in working hours of laborers due to environmental pollution and also offset the negative impact of health damages caused by environmental pollution on economic growth. It can also enhance the level of healthcare and the utilization of healthcare resources (21).

#### 3.1.4 Health, consumption, savings and economic growth

Consumption is a stable smoothing process across the life cycle of an individual. An increase in health status encourages individuals to boost their savings and material capital accumulation in their later years. Simultaneously, it also corresponds to higher expenditure on healthcare and other consumption goods, which can exacerbate the financial burden on younger generations and ultimately result in a long-term reduction in overall savings and economic growth rates (22). Environmental pollution-induced health damage frequently results in excessive healthcare spending and precautionary measures by households, leading to a crowding-out effect on physical investments (savings), thereby impeding capital accumulation and regional economic growth (23). On the one hand, improvements in health are reflected in the “behavioral effect” of increased life expectancy on economic growth, which fosters savings and growth. However, on the other hand, there is a dampening effect on savings and growth due to the rise in the total dependency ratio (2). Therefore, to assess the impact of consumption and saving on economic growth, it is necessary to consider the proportional relationship between consumption levels and saving rates, keeping other conditions constant.

In a region where the entire population is assumed to be within the labor force, the total consumption level of the population is denoted as *C*_0_, and the per capita consumption level is represented by *c*_0_, i.e.*c*_0_ = *C*_0_/*N*. Taking into account the health damage caused by environmental pollution and the impact of the age structure of the population, when the labor force population of the region stands at *L* and the overall social consumption level of the region equals *C*, we establish that the average consumption level of the non-labor force population in the region is equivalent to φ(φ > 0) times that of the labor force population. Therefore, the average consumption level of the non-labor force population in the region will be equal to φ*c*. Subsequently, we can determine the total consumption level using Equation 4.


(4) $$\begin{array}{l} C=c_{0}L+\varphi c_{0}\left(N-L\right) \end{array}$$

Additionally, a correlation exists between the level of total social consumption influenced by environmental pollution *C*, the proportion of the non-labor force population in the region η , and the total consumption when the population is entirely within the labor force category *C*_0_. This relationship is further expressed in Equation 5.


(5) $$\begin{array}{l} C=c_{0}L+\varphi c_{0}\left(N-L\right)=\left(1-\varphi +\varphi \eta \right)C_{0} \end{array}$$

Considering the concept of the “second demographic dividend,” the aging of the population will result in an elevation of savings levels and a decline in consumption rates. To simplify the analysis, let's assume that the entire population of a region is comprised of the workforce. There exists a negative linear relationship between the total consumption *C*_0_ and the proportion of non-labor population in region η . This relationship can be represented as $\frac{\partial C_{0}}{\partial \eta }=-a\left(a>0\right)$ i.e., $\frac{\partial C}{\partial \eta }=\varphi C_{0}-a\left(1-\varphi +\varphi \eta \right)$. The magnitude of this relationship depends on the balance between φ*C*_0_, which represents the consumption loss effect due to environmental pollution, and the savings effect generated by the labor force *a*(1 − φ + φ η). In essence, determining whether the “second demographic dividend” can counteract the negative impacts of environmental pollution-induced labor loss, such as health issues affecting savings, largely depends on the ratio between the labor loss savings effect and the consumption effect.

#### 3.1.5 Expenditures on health, health services, and economic growth

Public spending on medical and health services can fulfill the public's fundamental health needs and contribute to averting or delaying the health damage caused by environmental pollution. To mitigate the health impacts of environmental pollution, the government inevitably increases its investment in healthcare services to satisfy the population's health requirements (24). The detrimental effects of environmental pollution on health will exacerbate the economic burden of healthcare, leading to a crowding-out effect on savings, impeding enterprise capital accumulation and economic progress. However, it will also elevate the level of expenditure on healthcare services, fostering the cultivation of healthy human capital and the growth of the health industry, ultimately exerting a positive influence on economic expansion (25). Specifically, the health damage caused by environmental pollution has both positive and negative repercussions on economic growth. Environmental pollution-induced health damage can either decrease public healthcare expenditure through improved environmental quality (income effect) or increase the level of public healthcare expenditure (substitution effect), thereby influencing economic growth.

### 3.2 Mathematical modeling of the impact of environmental pollution on economic growth from a health damage perspective

**Figure 1:**
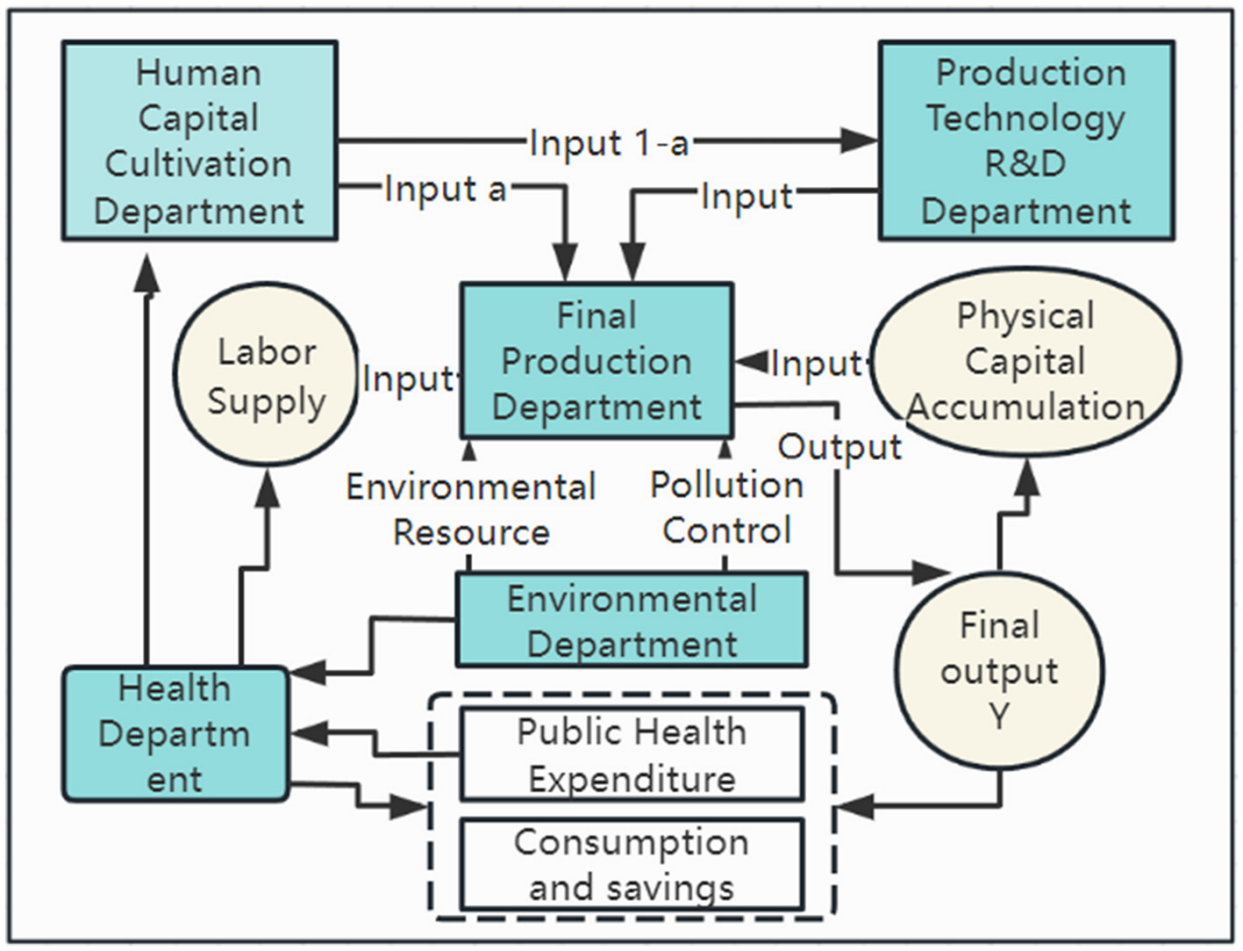
Internal growth model of five departments.

In this section, building on the mechanism analysis provided in the prior section and employing the framework of a four-sector endogenous economic growth model under environmental constraints, we will expand our theoretical exploration into the impact of health damage due to environmental pollution on economic growth. This will involve adding a health sector to our model, thus constructing a novel five-sector endogenous economic growth model that encapsulates the effects of environmental pollution on economic growth through the lens of health damage. The operating mechanism diagram is shown in Figure 1.

#### 3.2.1 Modeling

In a closed economic system, where consumers are also producers, the pursuit of consumer utility maximization is influenced by several factors. These include physical capital *X*, human capital *Y*, labor *X*, technological level *M*, and environmental resources *Y*. These elements are integrated into the production process, resulting in the creation of a single final product *M*. During production, the allocation of human capital is crucial. It is distributed between the technology research and development sector and the product production sector, following the Lucas (26) model. Specifically, the proportion of (1−*a*) and *a* is allocated to technology research and development, and product production, respectively, emphasizing innovation. Environmental resources, supplied by the environmental sector, are utilized during production activities. However, the production of final products results in the emission of environmental pollutants such as waste gas, wastewater, and waste residue. This emission subsequently imposes negative impacts on the ecological environment.

##### 3.2.1.1 Human capital development department

Health, as a crucial component of human capital, cannot be overlooked. Ignoring the impact of health on human capital accumulation could lead to biased regression outcomes. In the theoretical framework established in this article, concerning the detrimental effects of environmental pollution on economic growth, human capital remains a focal point. Specifically, the influence of health degradation due to environmental pollution is explored in relation to its effect on education levels, which directly impacts the efficiency of human capital accumulation (27). The model for human capital accumulation borrows from the conceptual approach of Lucas (26), suggesting that the level of human capital development is primarily determined by the existing stock of human capital and the efficiency with which it is cultivated (26). In the sector concerned with the development of human capital, efficiency is denoted as μ and the input ratio as *v*(0 < *v* < 1). The equation governing the accumulation of human capital within this sector can be articulated as follows:


(6) $$\begin{array}{l} \overset{•}{H}=\mu vH \end{array}$$

##### 3.2.1.2 Production technology research and development department

Drawing on He and Xu (28), this article formulates the output accumulation equation for the R&D sector as follows (28):


(7) $$\begin{array}{l} \overset{•}{A}=A_{T}{\left[\left(1-v-a\right)H\right]}^{\tau } \end{array}$$

Here, *A*_*T*_ represents the R&D efficiency parameter; (1−*v* − *a*)*H*, 0 < 1 − *v* − *a* < 1denotes the amount of human capital invested in the R&D sector; and 0 < τ represents the elasticity parameter of the amount of human capital invested, in relation to changes in the stock of technology level.

##### 3.2.1.3 Environmental department

Environmental pollution emissions represent one of the most prevalent negative externalities in business operations. Excessive emissions from production processes can lead to public health depletion and a reduction in welfare levels, adversely affecting regional economic growth (29). In this article, inputs of environmental resources, emissions of environmental pollution, and environmental governance are collectively considered under the umbrella of environmental sectors.

###### 3.2.1.3.1 Inputs of environmental resource factors

Inspired by the modeling approach of Stiglitz (30), we assume that environmental resource factors are non-renewable (30). The total per capita amount of the resource available at the initial point in time is denoted as *M*. The environmental sector develops and markets these resource factors to the production sector *c*′. The input of environmental resource factors varies with the production processes of manufacturers and over time, impacting the stock of environmental resources.

Assuming the cost of extracting environmental resources is 0, the change in the stock of environmental resources is *R* = *R*_0_ − ∫*R*(*t*)*dt*. At this juncture, the accumulation equation for *R* is presented.


(8) $$\begin{array}{l} \overset{•}{R}=-R \end{array}$$

###### 3.2.1.3.2 Environmental pollution emissions and environmental pollution control

Based on Zhao and Zhou's (31) research assumption, a firm's pollution emissions are directly proportional to its output, and the level of final production pollutant emissions is a function of the level of product output and the degree of government control over environmental pollutants (31). Of the various environmental resource elements, this paper focuses on the purification capacity of the ecological environment itself and the improvement of environmental pollution through government environmental regulation (32). We establish the environmental pollution flow equation as *P* = *Y*^*b*^*z*^−*r*^ where *P* represents environmental pollutant emissions, *b*(*b* > 0) denotes the production output of the final product sector, *b*(*b* > 0) represents the pollutant output elasticity, *z*(*z* ≥ 1) indicates the degree of pollution control. A stricter degree of control over environmental pollution emissions results in lower levels of environmental pollution emissions. *r*(*r* > 0) represents the elasticity of environmental pollution emission control, which reflects the efficiency of environmental pollution emission control, and the higher the value implies that the actual emission of pollutants to the environment is the less.

Additionally, the ecological environment system has a certain purification and absorption capacity for environmental pollutants. For the sake of simplicity in research, it is commonly assumed that this purification capacity is linear. This assumption is expressed as δ_*p*_*p*, where δ_*p*_(0 < δ_*p*_ < 1) (14). The stock accumulation equation of environmental pollution is then derived from these considerations:


(9) $$\begin{array}{l} \overset{•}{P}=Y^{b}Z^{-r}-\delta_{p}P \end{array}$$

##### 3.2.1.4 Production department

Drawing upon the Cobb-Douglas production function and referring to the research of He and Xu (28), the specific production function can be expressed as follows^1^ (28):


(10) $$\begin{array}{l} \text{Y}=AK^{\alpha }{\left(aH\right)}^{\beta }{\text{R}}^{\gamma }L^{1-\alpha -\beta -\gamma }z^{-1} \end{array}$$

In this context, 0 < α < 1, 0 < β < 1, 0 < γ < 1, 0 < α + β + γ < 1 denotes the output elasticity pertaining to physical capital *K*, human capital *H*, environmental resources *R*, labor *L*, and the technological productivity level of the production sector *A*. Furthermore, *z*(*z* ≥ 1) represents the intensity of government pollution control or environmental regulation. A higher value of *z* signifies a stricter implementation of pollution control (environmental regulation) by the government. It is posited that the level of government environmental control, *z* is inversely related to the output of the production sector *Y*. The depreciation rate of physical capital δ_*k*_, while $\overset{•}{K}$ signifies the per capita increase in physical capital. From this, the accumulation equation for physical capital *b* can be derived.


(11) $$\begin{array}{l} \overset{•}{K}=Y-\left(1-\varphi +\varphi \eta \right)C_{0}-\delta_{k}K-S \end{array}$$

##### 3.2.1.5 Health department

This article posits that the government's public health and hygiene inputs are denoted by *S*, which is derived from the total output *Y*. The stock of resources within the health sector is represented by *M*, while the rate of depreciation^2^ (encompassing factors such as the depreciation of aging medical devices and equipment, along with daily operation and maintenance costs) is denoted by δ_*M*_(δ_*M*_ > 0). $\overset{•}{M}$ signifies the incremental growth in resources for public health and hygiene services. χ represents the efficiency in transforming these inputs into public health and hygiene services. Consequently, the equation for the accumulation within the health sector can be expressed as:


(12) $$\begin{array}{l} \overset{•}{M}=\chi S-\delta_{M}M \end{array}$$

##### 3.2.1.6 Utility function

This research examines the repercussions of environmental pollution on health investments, postulating that the stock of public health expenditures, denoted by *M*, reflects the resource base within the health sector. The study introduces an additive isoelastic utility function that is contingent upon consumption, environmental pollution, and the standard of public health and sanitation services.


(13) $$\begin{array}{l} U\left(C,M,P\right)=\frac{{\left[\left(1-\varphi +\varphi \eta \right)C_{0}\right]}^{1-\varepsilon }-1}{1-\varepsilon }+\frac{M^{1-\omega }-1}{1-\omega }-\frac{P^{1+\psi }-1}{1+\psi } \end{array}$$

The constants ε, ω and ψ, each >0, quantify the respective influences of consumption, public health and sanitation expenditures, and environmental pollution on consumer utility. Assuming the existence of rational consumers within the society who aim to maximize the present value of societal utility in the current period, the objective is set accordingly. The total societal utility function, aspiring for an infinite temporal scope, is defined as: $U=\overset{\infty }{\underset{0}{\int }}U\left(C,M,P\right)e^{-\rho t}dt$,where ρ(ρ > 0) symbolizes the consumer time discount rate, illustrating consumer preference for present consumption over future consumption, and *e*^−ρ*t*^ serves as the discount factor.

The complete economic growth model, formulated through the establishment of the aforementioned objective function, is presented as follows:


(14) $$\begin{array}{l} \max\int_{0}^{+\infty }\left\{\frac{{\left[(1-\varphi +\varphi \eta )C_{0}\right]}^{1-\varepsilon }-1}{1-\varepsilon }+\frac{G^{1-\omega }-1}{1-\omega }-\frac{P^{1+\psi }-1}{1+\psi }\right\}e^{-\rho t}dt\\ \;s.t.\\ \text{ Y}=AK^{\alpha }(aH{)}^{\beta }{\text{R}}^{\gamma }L^{1-\alpha -\beta -\gamma }z^{-1};\overset{•}{\;K}=Y-\left(1-\varphi +\varphi \eta \right)\\ \;C_{0}-\delta_{k}K-S\\ \;\overset{•}{H}=\mu vH;\overset{•}{\;A}=A_{T}[(1-v-a)H{]}^{\tau }\;\overset{•}{R}=-R\\ \;\overset{•}{P}=Y^{b}z^{-r}-\delta_{p}P;\overset{•}{M}=\chi S-\delta_{M}M \end{array}$$

where*K, H, A, R, P, M* is the state variable and *C*_0_, *a, v, z, S* is the control variable.

#### 3.2.2 Model solving

Assuming the growth rates of all aforementioned variables remain constant, addressing the first-order conditions for achieving the optimal economic growth rate through Pontryagin's maximum principle effectively resolves the model's dynamic optimization dilemma, ultimately determining the steady-state growth rate of the economy. Given space constraints, the specific computational methods are omitted from this discussion. Let $g_{x}=\overset{•}{X}/X$ symbolize the growth rate of any variable *X*, then it follows that:


(15) $$\begin{array}{l} g_{P}=\frac{1-\varepsilon }{1+\psi }g_{Y}g_{z}=\left[\frac{\varepsilon -1+b\left(1+\psi \right)}{r\left(1+\psi \right)}\right]g_{Y}g_{H}=\left(1-\varepsilon \right)g_{Y}\\ \;+\mu -\rho \\ g_{A}=\tau \left(1-\varepsilon \right)g_{Y}+\tau \left(\mu -\rho \right);g_{M}=g_{S}=g_{K}=g_{Y}=g_{C_{0}};\\ \;g_{Y}=\alpha g_{K}+\beta g_{H}+\gamma g_{R}+\left(1-\alpha -\beta -\gamma \right)g_{A}\\ \;+\left(1-\alpha -\beta -\gamma \right)g_{L}-g_{Z} \end{array}$$

When the environmental resources in the ecosystem are assumed to be non-renewable with a growth rate of 0, i.e., *g*_*R*_ = 0, then there is:


(16) $$\begin{array}{l} g_{Y}=\\ \frac{r\left(1+\psi \right)\left\{\left(\mu -\rho \right)\left[\beta +\tau \left(1-\alpha -\beta -\gamma \right)\right]+\left(1-\alpha -\beta -\gamma \right)\left(n-\sigma \eta \right)\right\}}{\left(\varepsilon -1\right)+b\left(1+\psi \right)+r\left(1+\psi \right)\left\{\left(1-\alpha \right)-\left(1-\varepsilon \right)\left[\beta +\tau \left(1-\alpha -\beta -\gamma \right)\right]\right\}} \end{array}$$

At the same time, this article also derives the economic growth rate at steady state without considering the health effects of environmental pollution as:


(17) $$\begin{array}{l} g_{Y}=\\ \frac{r\left(1+\psi \right)\left\{\left(\mu -\rho \right)\left[\beta +\tau \left(1-\alpha -\beta -\gamma \right)\right]+n\left(1-\alpha -\beta -\gamma \right)\right\}}{\left(\varepsilon -1\right)+b\left(1+\psi \right)+r\left(1+\psi \right)\left\{\left(1-\alpha \right)-\left(1-\varepsilon \right)\left[\beta +\tau \left(1-\alpha -\beta -\gamma \right)\right]\right\}} \end{array}$$

To ensure the existence of an optimal path for economic growth in a stable state, it is imperative to achieve a favorable combination of conditions: a sustained positive economic growth rate, a decreasing rate of environmental pollution emissions, and an increasing cumulative rate of public health expenditures.

(1) The economic growth rate is positive when certain conditions are met. Specifically, to ensure this, we require *g*_*Y*_ > 0, *g*_*M*_ > 0, *g*_*P*_ < 0, and both ε > 1 and μ > ρ must be satisfied. ε represents the consumer's willingness to consume across different time periods. When ε > 1, it indicates that, over any two-time period, the elasticity of substitution in the consumer's product consumption (1/ε) fluctuates between 0 and 1. This preference restriction favors a reduction in the consumer's current consumption, encouraging a “smoothing” consumption pattern. This ensures that the material goods sector aligns with the regular production schedule, allowing for environmental resources to be consumed according to the normal production schedule. Consequently, pollutant emissions during production remain at a steady level, facilitating regularized control of environmental pollution by the environmental management sector, thereby maintaining sustainable economic growth.

Furthermore, μ > ρ indicates that, on the equilibrium growth path, various factors such as output, consumption, investment, physical capital, and public health and sanitation expenditures expand at the same growth rate, only if the accumulation of human capital exceeds the time discount rate. For sustainable output and consumption growth, the rate of human capital accumulation must exceed the economic growth rate. This ensures that human capital accumulation is sufficient to effectively counteract the negative externalities of environmental pollution and consumers' relatively “impatient” characteristics.

(2) Negative growth rate of environmental pollution. The negative growth rate of environmental pollution signifies a positive shift in environmental protection measures taken by manufacturers. Not only are they ensuring sustained economic growth but also mitigating the level of environmental pollution. Specifically, this indicates a decline in the growth rate of environmental pollution emissions, leading to a gradual improvement in environmental quality. Along the balanced growth path, as the environmental management department intensifies its focus on environmental protection, enterprises are compelled to adopt clean energy sources, bolster environmental protection technology upgrades, and invest in research and development. These efforts contribute to enhancing environmental quality.
(3) Positive rate of accumulation of public health resources. Furthermore, along the optimal path of economic growth, the rate of accumulation of public health resources demonstrates a positive trend. This is parallel to the economic growth rate, as exemplified by the positive value of public health and hygiene expenditure ratio. It suggests that enhancing the service efficiency of the public healthcare sector is conducive to boosting economic growth. Conversely, with an increase in the economic growth rate, there is a corresponding rise in the demand for basic public resources, such as healthcare and hygiene, both in quantity and quality. To meet the escalating demands for public health and hygiene resources, it becomes imperative for the government to augment its investment in these resources.

### 3.3 Comparative static analysis

To delve deeper into the impact of various factors on economic growth, this study employs Equation 16 to derive each parameter of the aforementioned expression. Through a comparative static analysis, the following conclusions are reached:

#### 3.3.1 Conclusion 1

Elevating levels of environmental pollution are detrimental to economic growth. Enhancing the efficiency of environmental pollution control and mitigating pollution levels can effectively drive economic growth. Research hypothesis 1 has been validated.

#### 3.3.2 Conclusion 2

Health damage resulting from environmental pollution negatively impacts the level of human capital accumulation, which is a significant factor in promoting economic growth. As stated in research hypothesis 1.

#### 3.3.3 Conclusion 3

Environmental pollution-induced health damage reduces the level of labor supply. Increasing the labor supply can effectively promote economic growth.

#### 3.3.4 Conclusion 4

Improving the efficiency of human capital transformation can enhance technological innovation and reduce environmental pollution, thereby promoting economic growth.

#### 3.3.5 Conclusion 5

Expenditures on public health and sanitation services alleviate health damage caused by environmental pollution. However, the impact of these expenditures on economic growth remains uncertain.

#### 3.3.6 Conclusion 6

Health damage caused by environmental pollution results in relative changes in labor consumption and savings levels. These consumption and savings are crucial transmission channels through which environmental pollution's impact on health affects economic growth. However, the influence of both on economic growth depends on their proportional relationship.

## 4 Empirical analysis

In this section, we build upon the production function established in the theoretical model discussed previously, modeling the corresponding equations to explore how the interplay between environmental pollution and health impacts economic growth, both before and after acknowledging health damages attributable to environmental pollution.

### 4.1 Impacts of environmental pollution and health on China's economic growth

#### 4.1.1 Model configuration and variable selection

This study integrates both health and education variables into the equation for economic growth to elucidate the respective impacts of healthy and educated human capital on economic growth, formulated as follows:


(18) $$\begin{array}{l} Y_{it}=\alpha_{0}+\alpha_{1}K_{it}+\alpha_{2}H\text{e}{\text{l}}_{it}+\alpha_{3}Edu+\alpha_{4}R_{it}+\alpha_{5}L_{it}+\alpha_{6}P_{it}\\ \;+\xi_{i}+{\text{λ}}_{t}+\varepsilon_{it} \end{array}$$

Where ξ is time fixed effect; λ is regional fixed effect; and ε is a random perturbation term.

To investigate the impact of environmental pollution on the contribution of healthy human capital to economic growth, this study extends Equation 19 by incorporating an interaction term between health and environmental pollution into the economic growth equation for empirical testing, represented by:


(19) $$\begin{array}{l} Y_{it}=\alpha_{0}+\alpha_{1}H\text{e}{\text{l}}_{it}+\alpha_{2}P_{it}+\alpha_{3}H\text{e}{\text{l}}_{it}\times P_{it}+\sum \alpha_{j}X_{ijt}+\xi_{i}\\ \;+{\text{λ}}_{t}+\varepsilon_{it} \end{array}$$

Moreover, considering the negative externalities associated with environmental pollution and acknowledging the rapid advancement of urban transportation within China, it is evident that convenient transportation significantly enhances regional population mobility. This advancement promotes more frequent and intimate regional cultural and economic interactions. Consequently, this article employs the Spatial Durbin Model to delve into the spillover effects between environmental pollution and the level of human capital. This approach facilitates a more detailed exploration of the impacts of environmental pollution and health levels on local economic growth in neighboring regions. The Dynamic Spatial Durbin Model constructed in this paper is as follows:


(20) $$\begin{array}{l} Y_{it}=\vartheta Y_{it-1}+\rho \underset{j\neq i}{\sum }W_{ij}\times Y_{it}+{\text{λ}}_{1}P_{it}+{\text{λ}}_{2}\underset{j\neq i}{\sum }W_{ij}\times P_{it}\\ +{\text{λ}}_{2}H\text{e}{\text{l}}_{it}+{\sum \text{λ}}_{j}M_{ijt}+\xi_{i}+{\text{λ}}_{t}+\varepsilon_{it} \end{array}$$

Regarding the spatial weighting term *W*_*ij*_, this study adopts an economic-geographical matrix that encompasses both geographical and economic distance factors. Prior to conducting spatial econometric regression analysis, the utilization of “Moran's *I*” reveals that the Moran's indices for economic growth level, the composite index of environmental pollution, and the composite index of health are all positive. These indices have largely been validated through significance testing, indicating a spatial interdependence and clustering by location.

Each main variable is characterized as follows:

1) Healthy human capital: it is an integral part of human capital. To accurately assess the health levels of residents in various cities in China, this article constructs a comprehensive measurement system for the level of health human capital (33). The specific indicator system is shown in Table 1.
In the measurement of comprehensive indicators, to ensure the cross-year comparability of the comprehensive index, the first step is to use 2002 as the base period, and then standardize the original data using the extreme value data standardization method. Subsequently, the longitudinal and horizontal differentiation method is employed for weighting. This method directly derives weighting information from the original data of the evaluated objects, and determines corresponding weights based on the amount of information provided by each indicator. It effectively avoids the influence of subjective preferences on the evaluation results, making the evaluation more intuitive and comparable among different evaluation objects and periods. This method is particularly suitable for panel data (34). Finally, the comprehensive index is obtained through linear weighting method.
2) To precisely measure the level of environmental pollution across Chinese cities, this study develops an environmental pollution level indicator measurement system (4). Similarly, the improved vertical and horizontal terms are used for weighting, with higher indicator values indicating more severe environmental pollution. The specific indicator system is shown in Table 2.
3) The remaining primary explanatory variables are as follows: the level of economic growth, which is determined by converting the nominal GDP of each city to real GDP, with the GDP index of 2002 as the base period; physical capital, represented by the capital stock as a measure of capital inputs; education human capital, indicated by the average number of years of schooling per capita; and the consumption of environmental resources, reflected by the total energy consumption of the region. In the empirical analysis, the GDP, physical capital, labor supply, and environmental resources were all processed with logarithmic transformation. Descriptive statistics for each variable can be found in Table 3.

**Table 1:** Evaluation system for comprehensive urban health indicators in China.

**Primary index**	**Secondary index**	**Tertiary index**	**Index description**	**Attribute**
Health fundamentals	Health status	Life expectancy	Population health level and survival time	+
		Mortality rate	Hygiene practices and quality of care	-
		Proportion of children under 5 years of age who are moderately malnourished	Dietary nutritional status and level of the population	-
		Incidence rate of statutorily reported infectious diseases in categories A and B	Evolution of infectious diseases	-
	Age structure	Older adult population dependency ratio	Level of population aging	-
Health security	Medical and health security	Hospitals per million population	Supply of medical institutions, level of access	+
		Number of practicing physicians per million population	Level of supply of health personnel per capita	+
		Hospital beds per 10,000 population	Scale of health hardware facilities	+
	Medical resource utilization	Bed occupancy rate in medical institutions	Extent of utilization of health resources	+
		Average hospital days	Medical benefits and technical level	-
		Average number of visits by the population	Extent of utilization of health resources	+
	Government investment	Total government investment in health as a share of GDP	Percentage of total government expenditure on health	+
	Personal investment	Total health costs per capita	Expenditures on personal health care	-

**Table 2:** Evaluation system for comprehensive indicators of environmental pollution level in Chinese cities.

**Primary index**	**Secondary index**	**Tertiary index**	**Index description**	**Attribute**
Pollution emissions	Industrial pollution	Total industrial wastewater discharge	Socio-economic development pressures on the water environment	+
		Total industrial dust emissions	Pressure on the atmospheric environment from socio-economic development	+
		Industrial solid waste generation	Pressure of socio-economic development on the ecosystem	+
		Total sulfur dioxide emissions	Pressure on the atmospheric environment from socio-economic development	+
	Domestic pollution	Domestic waste removal	Pressure on ecosystems	+
		Domestic wastewater discharge	Pressure on the water environment	+
		Equivalent level for environmental noise monitoring	Human health and the living environment	+
	Carbon footprint	Total CO_2_ emissions	Pressure on the atmospheric environment from socio-economic development	+
Pollution absorption	Natural absorption	Green space per capita	Living environment and quality of life	-
		Greening coverage rate of urban built-up areas	Degree of improvement in pollution through greening	-
		Average daily temperature	Impact on the removal of air pollutants	-
		Average annual precipitation	Impact on pollutant removal by suction	-
		Average urban relative humidity	Impact on climate	-
		Water resources per capita	Living environment and quality of life	-
	Environmental governance	Industrial wastewater discharge compliance	Industrial pollution control	-
		Industrial dust removal	Industrial pollution control	-
		Comprehensive utilization of industrial solid waste	Industrial pollution control	-
		Investment in environmental governance as a share of GDP	Government pollution control efforts	-
		Percentage of built-up area of smoke control areas	Environmental construction	-
		Amount of non-hazardous domestic waste disposed of	Domestic pollution control	-
		Domestic wastewater treatment capacity	Domestic pollution control	-
		Share of noise compliance area	Human health and the living environment	-

**Table 3:** Descriptive statistics for each variable.

**Variable**	**Symbol**	**Observed**	**Mean**	**SD**	**Min**	**Max**
Gross domestic product (GDP)	*Y*	5,985	1449.71	2375.41	17.828	30,633
Physical capital	*K*	5,985	2883.65	4389.79	40.125	57,059
Health human capital	*Hel*	5,985	0.535	0.074	0.343	0.791
Educational human capital	*Edu*	5,985	9.127	0.921	6.042	14.023
Labor force	*L*	5,985	48.721	77.962	4.051	1,980
Environmental resources	*R*	5,985	1171.75	1340.32	10.964	11,859
Environmental pollution	*P*	5,985	0.643	0.067	0.432	0.754

#### 4.1.2 Empirical results

For the regression analysis, panel data fixed effects were chosen based on the panel data Hausman test method. In instances where generalized method of moments (GMM) is applied for regression analysis, the Systematic Generalized Method of Moments (SYS-GMM) model is preferred due to its enhanced capability to address the issue of weak instrumentality (35). Consequently, this study primarily focuses on reporting the regression outcomes obtained through the SYS-GMM approach. Table 4 presents the regression results concerning the overall impact of environmental pollution and health on China's economic growth.

**Table 4:** Regression results of the overall effect of environmental pollution and health on China's economic growth.

**Dependent variable: Economic growth**	**Fixed effect**				**SYS-GMM**	**Space durbin**
	**(1)**	**(2)**	**(3)**	**(4)**	**(5)**	**(6)**
*L. GDP*					0.761^***^	1.451^***^
					(31.44)	(27.92)
*P*	−12.684^***^		−1.113^***^	−0.814^**^	−1.396^***^	−2.042^***^
	(−13.95)		(−3.23)	(−2.13)	(−3.25)	(−8.00)
*Hel*		2.468^***^	2.443^***^	3.854	4.034^***^	0.133^**^
		(6.28)	(6.06)	(0.97)	(6.62)	(2.05)
*Edu*		1.674^***^	1.674^***^	1.675^***^	0.852^***^	0.198
		(6.40)	(6.43)	(5.43)	(5.06)	(1.49)
*K*		0.198^***^	0.195^***^	0.298^***^	0.214^***^	0.182^***^
		(9.80)	(5.49)	(6.49)	(6.98)	(6.95)
*L*		0.093^***^	0.090^***^	0.167^***^	0.122^***^	0.087^***^
		(5.66)	(5.48)	(4.92)	(6.54)	(6.33)
*R*		0.721^***^	0.725^***^	0.631^***^	0.384^***^	0.309
		(6.16)	(5.31)	(6.44)	(8.78)	(1.05)
H^*^P				−0.151^***^		
				(−3.91)		
W^*^P						−1.928
						(−0.54)
W^*^H						−1.879^**^
						(−2.14)
*AR(1)* *AR(2)*					0.000	
					0.131	
*Hansen-P*					0.216	
*Spa-rho*						0.822^***^
						(19.80)
*Cons*	34.775^***^	−8.189^***^	−5.671^***^	−1.066	−7.021^***^	
	(17.24)	(−8.58)	(−3.02)	(−0.17)	(−9.01)	
*N*	5,985	5,985	5,985	5,985	5,700	5,700

^**^ and ^***^ indicate significance at the 5 and 1% levels, respectively; values in parentheses are z-values.

In Table 4, columns (1) to (4) detail the regression outcomes derived from the panel data fixed effects model. Specifically, column (1) provides the regression analysis focusing solely on the impact of environmental pollution variables on economic growth. The analysis revealed a statistically significant negative correlation between environmental pollution variables and economic growth, highlighting the detrimental effect of environmental pollution on economic growth. Specifically, with every unit increase in environmental pollution level, there will be a decrease of 12.684% in the level of regional economic growth. Column (2) explores the economic growth equation without accounting for environmental pollution levels, revealing a significant positive relationship between healthy human capital and economic growth. The present study indicates that healthy human capital has made substantial contributions to economic growth, with a one-unit increase in healthy human capital resulting in an economic growth increase of 2.468%. Research hypotheses 1 and 2 have been verified.

Column (3) integrates both environmental pollution and health levels into the regression equation affecting economic growth. The research results show that the coefficient between environmental pollution and economic growth remains significantly negative. Specifically, every increase of 1 unit in environmental pollution leads to a decrease of 1.113% in economic growth. Conversely, the coefficient indicating the impact of healthy human capital on economic growth remains significantly positive, but the regression coefficient between them has slightly decreased. Specifically, every increase of 1 unit in the level of healthy human capital contributes to an economic growth of 2.443%. This raises questions about whether the contribution of health to economic growth will decrease after incorporating the environmental pollution variable, and whether environmental pollution has an inhibitory effect on health status.

Column 4 introduces an interaction term between health levels and environmental pollution levels within the economic growth equation to assess whether environmental pollution modifies the health-related enhancement of economic growth. The negative coefficient of significance attached to this interaction term suggests that the beneficial impact of health on economic growth diminishes with an increase in pollution levels. This finding provides evidence in favor of Research Hypothesis 3, arguing that environmental pollution not only directly obstructs economic progress but also exerts an influence on individuals' health, subsequently mitigating the positive contribution of good health to economic growth. This phenomenon can be attributed to the increased financial investment in pollution control and health preservation by local governments in response to pollution-induced health detriments, leading to a “crowding-out effect” on economic production inputs, concurrently diminishing health's contribution to economic growth.

Column 5 presents the robustness check results using the generalized method of moments (GMM) for a dynamic two-stage system, aiming to affirm the reliability of the regression findings while addressing potential endogeneity concerns. The Hansen test yields a *p*-value of 0.216, which exceeds 0.1, thereby passing the validity test for the instrumental variables. In the Arellano-Bond test for autocorrelation of instrumental variables, the *p*-values for AR(1) and AR(2) are 0.000 and 0.131, respectively. This indicates the presence of first-order autocorrelation but not second-order autocorrelation, satisfying the requirements of the autocorrelation test. The concordance between the GMM results and those from the fixed-effects model (3) using panel data—barring coefficient magnitude differences—reiterates that environmental pollution continues to impede economic growth, whereas health levels positively influence economic expansion.

Column 6 explores how environmental pollution and health levels in neighboring cities impact local economic growth through the lens of the dynamic Spatial Durbin model. The analysis reaffirms that local pollution levels deter economic development, while healthy human capital remains a potent catalyst for local economic advancement. Interestingly, pollution levels in adjoining cities showed no significant effect on the economic growth of the focal area. Given that this research considers the net value of environmental pollution, which lacks the spatial mobility inherent to atmospheric pollutants, neighboring pollution did not significantly hinder local economic growth. However, the health status of neighboring regions exhibited a slight repressive effect on local economic dynamism. This can be attributed to the 'siphon effect,' where improvements in a region's health standards attract high-level talents and enterprises to neighboring districts, potentially depleting the local talent pool, thus inadvertently impacting local economic development negatively.

### 4.2 The impact of environmental pollution-induced health damage on China's economic growth

The empirical analysis conducted in Section 3.1 revealed that the contribution of health to economic growth diminishes as environmental pollution levels escalate. Concurrently, the adverse effects of environmental pollution on health are well-documented. This raises the question: Does health damage resulting from environmental pollution further impede economic growth? Previous studies investigating the impact of health damage due to environmental pollution on economic growth have adopted two primary approaches. The first approach, exemplified by Zhao et al. (14), involves adjusting the health level to represent the health status post-environmental pollution exposure as a specific percentage of the original health level (14). The second approach, as utilized by Wang et al. (2), amalgamates environmental pollution metrics with health indicators, constructing a system to quantify health damage attributable to environmental pollution (2). These methodologies, however, suffer from significant subjectivity, presupposing the inevitability of health damage from environmental pollution and, as a result, lack objectiveness and scientific rigor. To address these shortcomings, this study emulates the methodology of Qi and Lu (5), focusing on the influence of environmental pollution-induced health damage on economic growth (5). This is achieved through the development of a simultaneous equations model. Employing regression analysis within this framework allows for a nuanced examination of the endogeneity issue stemming from potential bidirectional causality between the dependent and independent variables.

#### 4.2.1 Model setting and variable selection

Retaining the economic growth equation as established in previous discussions, this study constructs additional models to explore the interactions between health, environmental pollution, and economic growth. Specifically, we introduce a health equation and an environmental pollution equation, succinctly represented as:


(21) $$\begin{array}{l} Hel_{it}=C_{2}+\beta_{1}P_{it}+\beta_{2}PY_{it}+\beta_{3}City_{it}+\beta_{4}M\text{ed}{\text{p}}_{it}+\beta_{5}E\text{d}{\text{u}}_{it}\\ \;+\beta_{6}PD_{it}+\beta_{7}G\text{in}{\text{i}}_{it}+\varepsilon_{it} \end{array}$$

Furthermore, to examine the influence of economic growth on environmental pollution, an equation is formulated as follows:


(22) $$\begin{array}{l} P_{it}=C_{3}+\varsigma_{1}Y_{it}+\varsigma_{2}{Y_{it}}^{2}+\varsigma_{3}R_{it}+\varsigma_{4}IS_{it}+\varsigma_{5}FDI_{it}+\varsigma_{7}TI_{it}\\ \;+\varsigma_{8}PD_{it}+\varepsilon_{it} \end{array}$$

Among the various factors, *PY* denotes the level of per capita economic growth; *City* denotes the level of urbanization; *M*edp represents the level of regional per capita healthcare expenditure; *E*du signifies the level of regional education. Furthermore, *PD* reflects the density of the urban population, expressed as the ratio of the total population at the end of the year to the city's area. Grossman and Krueger (6) demonstrated that the scale effect, structural effect, and technological effect in the economic growth process have an impact on environmental pollution (6). Consequently, industrial structure value is denoted by *IS*, with the proportion of the tertiary industry in GDP used as a metric. *FDI* indicates foreign direct investment, and the proportion of foreign investment in regional investment is employed. This foreign direct investment has both the “pollution halo” and the “pollution paradise” effects. Technological innovation, represented as *TI*, is measured by the number of patent applications. This metric is chosen because advances in technological innovation are instrumental in not only enhancing production processes and efficiency but also in fostering the optimization and upgrading of regional industrial structures. Consequently, this reduces the emission of environmental pollutants during economic growth, thereby improving environmental quality.

In this study, we have developed and integrated economic growth equations, health equations, and environmental pollution equations into a simultaneous equations model. This integrated model is then employed for regression analysis to investigate the interconnectedness of economic growth, health outcomes, and environmental pollution.


(23) $$\{\begin{array}{l} Y_{it}=\alpha_{0}+\alpha_{1}K_{it}+\alpha_{2}Hel_{it}+\alpha_{3}Edu_{it}+\alpha_{4}R_{it}+\alpha_{5}L_{it}\\ \;\;\;\;\;\;+\alpha_{6}P_{it}+\varepsilon_{it}\\ Hel_{it}=C_{2}+\beta_{1}P_{it}+\beta_{2}PY_{it}+\beta_{3}City_{it}+\beta_{4}Medp_{it}\\ \;\;\;\;\;\;\;+\beta_{5}Edu_{it}+\beta_{6}PD_{it}+\beta_{7}Gini_{it}+\varepsilon_{it}\\ P_{it}=C_{3}+\varsigma_{1}Y_{it}+\varsigma_{2}Y_{it}^{2}+\varsigma_{3}R_{it}+\varsigma_{4}IS_{it}+\varsigma_{5}FDI_{it}\\ \;\;\;\;\;\;\;\;+\varsigma_{7}TI_{it}+\varsigma_{8}PD_{it}+\varepsilon_{it} \end{array}$$

#### 4.2.2 Empirical results

To assess the overarching impact of health detriments arising from environmental pollution on China's economic growth, this study employs the weighted two-stage least squares (2SLS) method for solving the simultaneous equations model. Notably, the weighted 2SLS method addresses potential heteroskedasticity issues within panel data, ensuring that the estimation results are both unbiased and consistent. The outcomes of this regression analysis are presented in Table 5.

**Table 5:** Regression outcomes for the interaction between environmental pollution, health, and economic growth.

**Economic growth**		**Health**		**Environmental Pollution**	
**(1)**		**(2)**		**(3)**	
*P*	0.879^***^	*P*	−0.317^***^	*GDP*	0.345^***^
	(11.04)		(−5.80)		(7.07)
*Hel*	2.428^***^	*PGDP*	0.001	*GDP^2^*	−0.025^***^
	(6.65)		(1.56)		(−5.38)
*Edu*	0.093^**^	*Edu*	0.079^***^	*R*	0.028^***^
	(2.02)		(13.09)		(6.96)
*K*	0.599^***^	*Gini*	−0.098^**^	*Is*	−0.001^***^
	(5.88)		(−2.15)		(−5.94)
*L*	0.390^***^	*City*	0.026^***^	*Fdi*	−0.004^***^
	(7.59)		(7.16)		(−6.16)
*R*	0.152^***^	*Medp*	0.068^***^	*Ts*	0.001
	(4.51)		(3.49)		(1.61)
*Cons*	−1.476	*PD*	−0.101^***^	*PD*	0.001
	(−1.57)		(−4.71)		(1.33)
		*Cons*	7.68^***^	*Cons*	2.439^***^
			(5.60)		(7.76)

^**^ and ^***^ indicate significance at the 5 and 1% levels, respectively; values in parentheses are z-values.

The regression outcomes of the economic growth equation (Model 1) reveal that, upon integrating the interplay between environmental pollution, health, and economic growth, environmental pollution no longer serves as a significant impediment to economic growth; conversely, it appears to foster economic expansion to a certain degree. Moreover, healthy human capital continues to substantially bolster economic growth. These findings diverge from previous research, suggesting that once the health detriments attributed to environmental pollution are accounted for, alongside the reciprocal relationship between environmental pollution and economic growth, the adverse effects of environmental pollution on economic growth are primarily manifested through health impairments rather than direct economic inhibition. This is because the escalation of environmental pollution emissions during the production process signifies an increase in the input of environmental resources, implying that greater inputs lead to enhanced outputs. This phenomenon aligns with China's historical development strategy, which involved trading significant environmental resource consumption for high economic growth rates (36). According to the Environmental Kuznets Curve (EKC) hypothesis, developing countries during their initial industrialization phase often prioritize manufacturing industries with high pollution and high value-added (6). While the expansion of production undoubtedly leads to increased resource use and pollution emissions, it could also positively influence economic growth through various mechanisms such as factor-driven growth, economies of scale, heightened market competitiveness, cost reduction strategies, externalization of environmental costs, and the multiplier effect. At this juncture, healthy human capital retains its capacity to significantly drive economic growth, indicating that the current extent of health damages due to environmental pollution in China has not reached a severity that entirely negates the positive impact of health on economic growth. The contribution of healthy human capital to economic growth can still compensate for the economic losses incurred from health damages due to environmental pollution. Research hypotheses 3 has been verified.

The regression results of the health equation (Model 2) demonstrate that the level of environmental pollution significantly undermines health status, corroborating the health damage effects induced by environmental pollution. This finding is consistent with those of existing research, affirming the detrimental consequences of environmental pollution on health.

Drawing on the Environmental Kuznets Curve (EKC) hypothesis and evidence from prior studies indicating that the turning points between environmental pollution and economic growth vary by region and pollutant type, this study incorporates the quadratic term of economic growth into the environmental pollution equation (1, 6). This approach aims to examine whether a similar non-linear relationship exists between comprehensive ecological quality and economic growth. The regression outcomes of Model (3) analyzing environmental pollution reveal that there is a discernibly positive correlation between the primary coefficient of economic growth and environmental pollution levels. Conversely, the secondary coefficient of economic growth and the level of environmental pollution exhibit a significantly negative correlation. This pattern delineates the relationship between economic growth levels and environmental pollution as an inverted U-shaped curve, orienting downwards. Calculations pinpoint the curve's inflection point at a highly improbable high-income level, potentially due to utilizing a measured net environmental pollution index in this study. Consequently, this suggests that China's current economic growth level has not yet approached the inflection point, with environmental pollution levels still escalating in tandem with the economy's growth. Moreover, the regression analysis indicates that environmental resource consumption markedly elevates pollution levels. In contrast, technological innovation's impact on pollution levels is statistically insignificant. Both industrial upgrading and foreign investments contribute effectively to mitigating environmental pollution levels, whereas population density's influence on pollution remains statistically negligible.

## 5 Conclusions and recommendations

### 5.1 Conclusions

In the theoretical segment of our analysis, via the construction of a five-sector endogenous growth model that incorporates environmental pollution, we made several key findings: (1) Environmental pollution impacts economic growth both directly and indirectly. The indirect effects propagate through various channels including human capital accumulation, labor supply, technological innovation, consumption and savings, as well as public health expenditures. (2) Environmental pollution tends to reduce the economy's steady-state growth rate. Strategies to mitigate this include improving human capital levels and its accumulation efficiency, increasing labor supply, enhancing technological innovation, optimizing the use of environmental resources, bolstering the effectiveness of pollution control measures, and scaling reasonable public health spending. These efforts collectively aim for a “win-win” scenario of sustained economic growth coupled with reduced environmental pollution. (3) A critical oversight in considering environmental pollution's impact on economic growth is the neglect of health-related damages. Failing to account for health impairments can lead to underestimating both the severity of environmental pollution and its adverse effects on economic growth, while simultaneously overestimating the steady-state economic growth rate.

Through the empirical analysis conducted by establishing the model, we found: (1) Upon an isolated examination of the influence of environmental pollution and health on economic growth, it has been discovered that environmental pollution obstructs economic progress, whereas a healthy state effectively stimulates economic growth. Furthermore, environmental pollution diminishes the positive impact of good health on economic growth. (2) Upon examining the impact of health detriments caused by environmental pollution on economic growth, it was discovered that environmental pollution no longer directly impedes economic growth and may even foster it to a certain extent. The adverse effects of environmental pollution are more pronounced in its detrimental impacts on health. Enhancing health can effectively stimulate economic growth in various cities.

### 5.2 Policy recommendations

(1) Establishing a new development concept, enhancing environmental governance efficiency, and promoting green and sustainable economic growth. To mitigate the level of environmental pollution and foster green and sustainable economic growth, it is imperative to firmly establish the development concept of “innovation, coordination, greenness, openness, and sharing.” Recognizing the detrimental impact of environmental pollution on population health and its negative effects on economic growth, it is necessary to base decisions on the second national census of pollution sources. The government's leading role in environmental governance should be fully leveraged to standardize cooperation among local governments, coordinate market-oriented and trans-regional environmental policies, establish and refine multi-modal coordination mechanisms for environmental cooperation. Moreover, it is crucial to enhance the responsibility for resource and environmental regulation.

(2) Mitigating the indirect impact of environmental pollution-induced health damage on economic growth and contributing to sustained economic growth. The government should prioritize cultivating high-level talents, enhancing the efficiency of human capital accumulation, and fostering a favorable environment for human capital growth to elevate the level of labor supply and the general wellbeing of the workforce. Additionally, it is essential to establish a governance system for human resources development, guide labor mobility, and allow the market mechanism to play its pivotal role in resource allocation. The equalization of regional healthcare allocation should be coordinated to improve health protection measures for the workforce, promote the growth of the health industry, shape new economic growth points, and drive high-quality development within China's economy and society.

## Data Availability

The original contributions presented in the study are included in the article/supplementary material, further inquiries can be directed to the corresponding author.

## Footnotes

^1^For ease of writing, all equations in this paper omit subscripts.

^2^Medical resources include many, of which the number of medical and health institutions, the number of beds and the number of personnel in health institutions are three major and representative medical resources, and thus depreciation is considered mainly from these three aspects.
